# Trap-effectiveness and response to tiletamine-zolazepam and medetomidine anaesthesia in Eurasian wild boar captured with cage and corral traps

**DOI:** 10.1186/1746-6148-9-107

**Published:** 2013-05-23

**Authors:** José Angel Barasona, Jorge Ramón López-Olvera, Beatriz Beltrán-Beck, Christian Gortázar, Joaquín Vicente

**Affiliations:** 1 Sanidad y Biotecnología (SaBio), Instituto de Investigación en Recursos Cinegéticos (IREC; CSIC – UCLM – JCCM), Ronda de Toledo, Ciudad Real s.n. 13005, Spain; 2 Servei d’Ecopatologia de Fauna Salvatge (SEFaS), Universitat Autònoma de Barcelona (UAB), Barcelona, Bellaterra E-08193, Spain

**Keywords:** Anaesthesia, Capture, Medetomidine, Stress, Tiletamine, Zolazepam, Wild boar

## Abstract

**Background:**

Capture, handling and chemical restraint are basic techniques often needed for research or management purposes. The aim of this study was testing a combination of tiletamine-zolazepam (TZ) (3 mg/kg) and medetomidine (M) (0.05 mg/kg) on Eurasian wild boar (*Sus scrofa*). A total of 77 free-ranging wild boar were captured by means of portable cages and corral traps and then anaesthetized with intramuscular darts using a blowpipe. The individual response to chemical immobilization was characterized using anaesthetic, clinical, and serum biochemical variables. After the procedure, 14 of these wild boar were monitored for 20 days using GPS-GSM collars.

**Results:**

Pre-release mortality during capture and handling (6.5%) was associated with severe trauma in corral traps. Capture specificity for wild boar was 96.3% and trapping effort was 16.5 days per captured wild boar. Mean induction period was 4.5 ± 2.2 min, hypnosis period enabling effective handling was 61.6 ± 25.4 min, and recovery period was 12.8 ± 12.1 min. No heart or respiratory failure due to added stress occurred and post-release monitoring by GPS-devices revealed no mortality due to anaesthesia. According to the best statistical model obtained, the main factor driving anaesthetic efficacy and stress indicators is trap type.

**Conclusions:**

Both cage and corral traps are efficient methods to capture wild boar. Cage traps are safer, as demonstrated by mortality rates as well as anaesthetic, physiological, and serum biochemical responses. This anaesthetic protocol is useful for prolonged handling of wild boar and allows sampling and collecting data for ecological and epidemiological studies.

## Background

Social sensitivity regarding environmental issues, animal health and animal welfare has increased worldwide [1,2]. These issues must be addressed when implementing research and management of wild ungulates [3,4], which usually include trapping free ranging animals. The Eurasian wild boar (*Sus scrofa,* L. 1758) is one of the terrestrial mammals with the broadest geographic range [5,6], and has an ecological, health and economic impact [7-9]. Several capture and handling studies, mostly using baited box traps and corral traps, have been carried out on this species [10-12]. Capture and either physical or chemical immobilization of wild boar convey risks of mortality, but chemical immobilization is usually required for handling [13-17]. Different factors, such as capture method, previous human-induced stress and environmental conditions may affect the efficacy of chemical restraint [18,19] and induce severe stress [14,17,19].

The most commonly drugs used in chemical immobilization of wild pig species are cyclohexamines (ketamine and tiletamine) and α_2_-adrenergic agonists (xylazine, X; detomidine; romifidine; medetomidine, M) [15-17,20,21]. Cyclohexamines are anaesthetics that cause electroencephalographic dissociation of the activity of the central nervous system, inducing visceral analgesia combined with superficial anaesthesia, persisting palpebral, laryngeal and pedal reflexes [22,23]. The α_2_-adrenergic agonists provide sedation, visceral analgesia and muscle relaxation [24-26]. The combination of these two types of drugs allows using lower doses to achieve hypnosis, analgesia and muscle relaxation [27]. The combination of α_2_-adrenergic agonists (M or X) and an opioid (morphine derivates) decreases the dose of the main anaesthetic, either propofol, thiopental, tiletamine or alfaxalone. This also minimizes the adverse effects of drugs used alone, e.g. agitated and violent recoveries in collared peccaries (*Tayassu tajacu*) anesthetized only with ketamine [13,28].

Several studies have assessed the usefulness of anaesthetic combinations to immobilize physically captured wild pig species [15-17,20,21]. The anaesthetic combination chosen in this study has been described for prolonged surgical procedures (high doses) by [20] (5 mg/kg of tiletamine-zolazepam, TZ and 0.1 mg/kg of M in 8 wild boar) and [29] (5 mg/kg of TZ and 0.025 mg/kg of M in 9 farmed will boar). Although serum biochemistry is a valuable tool to assess the physiological status of wild animals and the effect of handling and treatments, there is scarce knowledge on serum biochemistry values in wild boar [30-33], and few studies have investigated the physiological effects of anaesthesia after physical capture in this species [34].

The aims of this study were (1) to evaluate the efficiency and safety of a combination of TZ and M in free-ranging wild boar captured by means of cage and corral traps; and (2) to determine the factors affecting the anaesthetic and physiological individual response to chemical immobilization of wild boar physically captured with cage and corral-traps, using anaesthetic, clinical, and serum biochemical variables.

## Methods

### Study area and period

The study was conducted between February and November 2010 in Montes de Toledo (39° 25′ to 39° 16′ N, 4° 05′ to 4° 23′ W), in the region of Castilla-La Mancha, South-central Spain. This is a 36,000 hectares area where altitude ranges between 590 to 1010 meters a.s.l. Climate is Mediterranean, with an average temperature of 14.1°C. The habitat is characterized by evergreen oak (*Quercus ilex*) scrublands with scattered pastures and small crops, conforming *dehesas* (savannah-like habitats).

### Capture method

Seven 3 x 1.2 meters portable cage traps [10] and seven corral traps each consisting of seven panels over 5 meters wide [11] were used to capture wild boar. The portable cage traps were triggered when a wild boar stepped on a mobile bottom platform in the centre of the trap, which closed simultaneously the two drop gates of the trap. The corral traps had a single drop door and a trigger mechanism of root sticks. Traps were baited with corn every 2–4 days both inside and outside the trap and monitored with camera traps (Model IR-3BU, Leaf River Outdoor, Taylorsville, Mississippi, USA) to determine the time of activation. Once activated, each trap was revised daily, early in the morning to avoid that the animals reached high temperatures within the traps (maximum temperature in daytime reached up to 36.9°C). Study procedures were approved by the Animal Experiment Committee of Castilla-La Mancha University and were designed and developed by scientists (B and C animal experimentation categories) approved by the Spanish Ethic Committee.

### Anaesthesia and monitoring

A combination of TZ (Zoletil® 100 mg/ml, Virbac, France, target dose 3 mg/kg) and M (Medetor®, Virbac, France, target dose 0.05 mg/kg) was injected intramuscularly in the femoral region with 5 ml anaesthetic darts (Telinject®, Römerberg, Germany) using a 14 mm diameter blowpipe (Telinject®, Römerberg, Germany), after visually estimating the weight of each animal in the trap. After sedation, wild boar were removed from the traps and blindfolded.

Heart rate and oxygen saturation were measured and registered every 5 minutes from hypnosis to recovery using a portable modified pulse oximeter (G1B Pulse Oximeter, Quirumed®, Moncada, Spain). Respiratory rate was measured every 10 minutes by the same person by direct observation of chest wall movements. Rectal temperature was measured with a digital thermometer every 10 minutes. The following anaesthetic periods were registered: human presence until injection (HPI; from human arrival to the cage to injection time); induction period (IP; from injection to the loss of palpebral reflex and the possibility of handling); hypnosis period (HP; from loss of response to first movement and response to stimuli), and recovery period (RP; from first response to total coordination, walking without ataxia).

The wild boar captured were classified as juveniles (<24 months of age) or adults (>24 months of age) based on the eruption of molars and premolars [35,36]. Weight was measured with a scale, and total length (from snout to tail base) and thoracic perimeter were measured with measure tape and registered.

Blood was obtained from the ophthalmic sinus at the medial angle of the eye behind the nictitating membrane [37]. Blood samples were immediately refrigerated and transported to laboratory, within two hours after collection, where serum and plasma were obtained for biochemistry and blood smears were prepared.

### Serum biochemistry

Serum alanine aminotransferase (ALT), alkaline phosphatase (AP), aspartate aminotransferase (AST), creatine kinase (CK), and lactate dehydrogenase (LDH) activities and serum lactate, glucose, cholesterol, triglycerides, urea, creatinine, sodium, and potassium concentrations were determined by means of an automated analyser (Olympus AU400, Olympus, Tokyo, Japan).

### Post-release monitoring

Collars provided with a satellite position capture system (GPS) [38] and a global system for mobile communications (GSM) [39] were fitted to 14 wild boar over 40 kg. The collars were set to record one position every hour, sending encoded packets with 20 positions to the central station when mobile phone coverage was sufficient. Activity patterns were calculated for each collared animal by estimating the average speed obtained from the distance between two consecutive GPS locations. Post-capture monitoring was performed for 20 days. Collarless wild boar were ear-tagged. In addition, all the wild boar were identified with a microchip (FDX-B transponders, Allflex®, France) placed caudal to the ear. The entire procedure lasted less than 20 minutes in all cases. The body surface of the wild boar was wetted with cold water prior to release in order to avoid hyperthermia when rectal temperature exceeded 40°C.

### Statistical analysis

Descriptive statistics were calculated for physiological variables and anaesthetic periods (Statistica 7, Statsoft®, Tulsa, USA). In order to compare the number of wild boar captured per trap type and the GPS activity patterns (average speed) during the day post-release (10 hours after) against the 3 successive monitoring days, Mann–Whitney’s U test was used. Identity link generalized linear models (GLMz) [40] were carried out to explain the dependent variables: anaesthetic periods (IP, HP and RP), physiological (body temperature, respiratory and heart rates), and biochemical (ALT, ALP, CK, AST, LDH, lactate, glucose, cholesterol, sodium, potassium, urea, creatinine, triglycerides and total proteins) values (SPSS Statistics 18 for Windows, IBM®, Armonk, USA). In case wild boar were recaptured only the first measure was used to build the GLMz. Also individuals presenting trauma or obvious cachexia likely due to generalized tuberculosis were not included in the models. Wild boar injected with α_2_-antagonist atipamezole (Antisedant® 5 mg/ml, Orion Pharma, Finland, target dose 0.20 mg/kg) were excluded from the statistics. Serum enzymatic activities, which showed exponential variations, as we all the length of IP, HP, and RP were logarithmically transformed, in order to avoid overdispersion. The categorical independent variables were sex (1 = male, 2 = female), age class (1 = juvenile, 2 = adult) and type of trap (1 = cage trap, 2 = corral trap). Independent variables included as covariates were anaesthetic dose, body condition (as chest circumference to body length ratio) and maximum environmental temperature recorded the day of capture.

## Results

A total of 80 animals were captured: 77 wild boar, two adult badgers (*Meles meles*) and one yearling red deer (*Cervus elaphus*). Therefore, capture specificity for wild boar was 96.3%. The first capture took place on average 28.2 ± 8 days (range = 15–45) after starting baiting the trap. Each capture required on average 9 ± 3.6 visits to the trap (range = 4–16). Considering the baiting and trapping periods altogether, the average trapping effort was 16.5 ± 12.7 days per captured wild boar (Table 1).

**Table 1:** Capture data for the two physical capture methods (cage-traps and corral-traps) used in this study (adapted from López-Olvera et al. 2009)

**Capture method**	**Number of traps**	**Days before activation (mean; range)**	**Days activated (mean; range)**	**Days of work**	**Person-days of work**	**Number of capture events**	**Number of multiple captures**	**Wild boar captured**	**Wild boar per capture (mean; range)**	**Mortality**	**Days per wild boar (mean; range)**	**Person-days per wild boar (mean; range)**	**Gender and age class (Y=yearling; A=adult)**			
													**Male**		**Female**	
													**Y**	**A**	**Y**	**A**
Cage-trap	7	26; 11-44	1.8; 1-5	103	135	13	5	25	1.9; 1-5	1	21.8; 2-46	7.9; 1-16	7	8	6	4
Corral-trap	7	28.9; 15-45	2.1; 1-3	114	154	11	9	52	4.7; 1-11	4	10.2; 4-33	4.8; 2-16	22	8	18	4
TOTAL	14	27.4; 11-45	2; 1-5	217	289	24	14	77	3.2; 1-11	5	16.5; 2-46	6.5; 1-16	29	16	24	8

Pre-release mortality during capture and handling was 6.5% (n = 5); four deaths were associated with severe trauma (and subsequent euthanasia) in corral traps and one was due to hyperthermia (haemorrhages and cervical-thoracic congestion at necropsy) in a cage trap. The number of wild boar captured per trapping event was significantly higher for corral traps (4.73 ± 3.46) than for cage traps (1.92 ± 1.38) (Mann–Whitney U test; U = −2.36, *p* = 0.02) (Table 1). Capture selectivity by trap, gender, and age is also detailed in Table 1.

Out of the 77 captured wild boar, 42 were anaesthetized and 35 (weighing less than 15 kg) were handled without using chemical immobilization [41]. The mean dose used per anaesthetized wild boar was 2.9 ± 0.39 mg/kg of TZ and 0.048 ± 0.006 mg/kg of M. This was an 11% deviation from the target dose, due to errors in weight estimation. Double injection was needed only in one very excited and aggressive wild boar captured in a corral trap. Therefore, anaesthetic efficiency (percentage of fully anesthetized wild boar with a single injection) was 97.6%. For the 41 wild boar that received single injections, IP was 4.5 ± 2.2 min., HP enabling effective handling was 61.6 ± 25.4 min. and RP was 12.8 ± 12.1 min. The α_2_-antagonist atipamezole injected intramuscularly in 4 wild boar (40 min after anaesthetic drug injection) was effective in reversing immobilization, with recovery (total coordination) occurring 8.4 ± 2.3 min.

**Figure 1:**
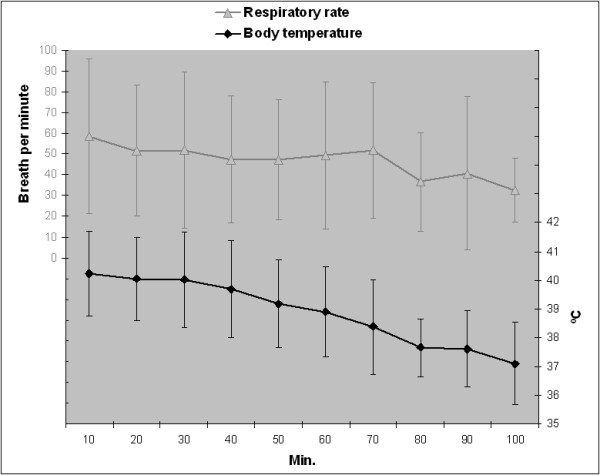
Mean ± SD respiratory rate and body temperature every 10 min.

**Figure 2:**
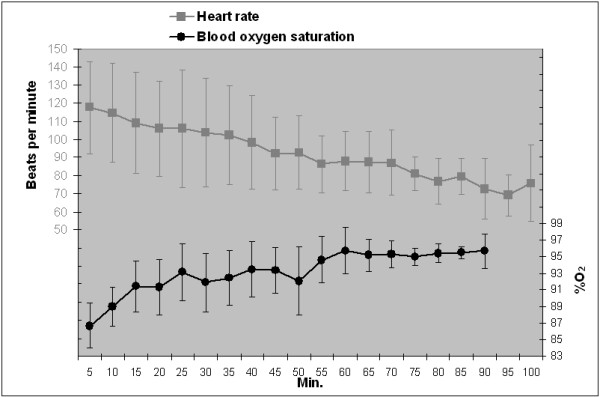
Mean ± SD oxygen saturation and heart rate every 5 min.

Figures 1 and 2 show average values and trends for respiratory rate and body temperature, and for heart rate and blood oxygen saturation, respectively. Respiratory rate, heart rate and rectal temperature decreased progressively, whereas oxygen saturation increased from the beginning of monitoring and remained stable between 90 and 96% SpO_2_ thereafter. No signs of vagal cardiorespiratory depression after drug administration were detected, but one wild boar presented transient atrio-ventricular arrhythmia during the deep anaesthetic phase. Body temperatures above 40°C were exceeded by 18 (47%) wild boar, of which 55% were males and 72% juveniles, mainly at the beginning of the anaesthesia.

After discarding recaptured wild boar (n = 1) and those presenting trauma (n = 5) or severe generalized tuberculosis (n = 2), the remaining 35 wild boar were included in the models. Table 2 shows the results of the GLMz and the value for each categorical variable to explain anaesthetic periods and biochemical variables. Tables 3 and 4 show the values for these variables.

**Table 2:** Results of Generalized Linear Models to identify factors associated variations in the anaesthetic periods, of the association between parameter estimator (β) for GLMz where in categorical variables “gender” “age class” and “type trap” the reference value of the parameter estimator was 0 for gender “female”, age class “yearling” and type trap “cage trap”

**Dependent variables**	**Independent variables**						
	**Trap type**	**Gender**	**Age class**	**Dosage**	**Body condition**	**Environmental temperature**	**Deviance difference**
**Log induction period**	−0.41***		−0.28**		−1.61**	0.01*	0.65
**Log hypnosis period**	0.30*						0.31
**Log recovery period**			−0.6***	0.49**			0.62
**Initial respiratory rate**				0.21*	−1.81**		0.24
**40 min respiratory rate**							0.59
**Initial heart rate**							0.26
**40 min heart rate**	−0.08*				−0.48*		0.36
**Initial body temperature**	−1.17*					0.10**	0.78
**40 min body temperature**							0.74
**Log ALT**				−0.27**			0.57
**Log ALP**			0.24*	−0.44***			0.63
**Log CK**				−1.17***	3.85*		0.62
**Log AST**		−0.29*		−0.62***			0.62
**Log LDH**				−0.62***	2.259*		0.59
**Lactate**	−0.13*			−0.18**			0.24
**Glucose**	−0.27***				−1.18**		0.72
**Cholesterol**						−0.05**	0.64
**Sodium**						0.002*	0.29
**Potassium**	−4.53*					−0.40**	0.59
**Log urea**				−0.19*		0.01*	0.28
**Creatinine**		−0.6*	−0.16***			0.01*	0.64
**Triglycerides**	−0.34***			−0.34**			0.54
**Total protein**		−0.35*	−0.04**				0.45

Body condition was measured as chest circumference to body length ratio. The proportion of explained deviance (Deviance difference) in each model is shown. P values are shown: * p < 0.05, ** p < 0.01, ***p < 0.001.

**Table 3:** **Mean values and reference ranges for the anaesthetic, physiological, and serum biochemical variables analyzed in 35 physically captured and anaesthetized wild boar ( *Sus scrofa *)**

	**N**	**Mean**	**SD**	**Range**	**Reference ranges**
**Induction period (min)**	35	**4.81**	2.92	13.16	N.A.
**Hypnosis period (min)**	32	**61.67**	25.36	106.12	N.A.
**Recovery period (min)**	30	**14.25**	13.41	46.5	N.A.
**Initial respiratory rate (/min)**	35	**61.74**	40.52	132	32-58*
**40 min respiratory rate (/min)**	32	**48.47**	31.96	146	32-58*
**Initial heart rate (/min)**	35	**114.94**	27.75	116	70-120*
**40 min heart rate (/min)**	31	**99.26**	26.26	118	70-120*
**Initial body temperature (°C)**	35	**40.27**	1.52	5.5	38.70-39.80*
**40 min body temperature (°C)**	31	**39.77**	1.77	5.9	38.70-39.80*
**ALT (UI/L)**	35	**64.91**	28.18	129	38-153.70
**ALP (UI/L)**	35	**111.15**	57.70	244.5	45.60-122.50
**CK (UI/L)**	35	**11,675**	36,788	216,811	918-3,106
**AST (UI/L)**	35	**260.09**	442.42	2180	52.30-113.40
**LDH (UI/L)**	35	**2805.78**	1159.14	4752.3	791-976
**Lactate (mmol/L)**	35	**13.44**	5.28	27.1	22.05
**Glucose (mmol/L)**	35	**8.64**	4.31	18.35	5.26-10.70
**Cholesterol (mmol/L)**	35	**2.78**	0.67	2.53	2.08-3.95
**Sodium (mmol/L)**	35	**148.14**	10.90	44.8	145.80-162.80
**Potassium (mmol/L)**	35	**11.93**	5.27	23.61	5.50-15.20
**Urea (mmol/L)**	35	**4.95**	1.46	6.79	2.40-5.25
**Creatinine (μmol/L)**	35	**128.68**	41.21	140.55	118.46-216.50
**Triglycerides (mmol/L)**	35	**0.41**	0.56	3.31	0.98-1.44
**Total protein (g/L)**	35	**78.50**	11.05	47.5	68.10-82.10

Wild boar reference values are provided by mean ranges from [33,42-44]. *Pig reference values adapted from [45].

**Table 4:** Value means (observed values / GLMz predicted values) for the anaesthetic, physiological, and serum biochemical variables showing statistically significant differences according to the independent categorical variables trap type, age class, and gender

**TRAP TYPE**	**Cage-trap**		**Corral-trap**	
**Hypnosis period (min)**	**74.79** / 72.63		**49.60** / 45.46	
**40 min heart rate (/min)**	**84.89** / 92.86		**96.71** / 103.54	
**Initial body temperature (°C)**	**39.96** / 39.96		**40.54** / 40.42	
**Lactate (mmol/L)**	**11.60** / 11.59		**15.15** / 15.24	
**Glucose (mmol/L)**	**6.63** / 6.63		**9.92** / 9.94	
**Potassium (mmol/L)**	**9.22** / 9.22		**13.37** / 13.63	
**Triglycerides (mmol/L)**	**0.21** / 0.21		**0.59** / 0.62	
**AGE CLASS**	**Young**		**Adult**	
**Recovery period (min)**	**10.28** / 7.38		**22.18** / 23.11	
**ALP (UI/L)**	**134.91** / 126.04		**74.68** / 64.76	
**GENDER**	**Female**		**Male**	
**AST (UI/L)**	**251.83** / 153.66		**279.44** / 186.33	
**TRAP TYPE**	**Cage-trap**		**Corral-trap**	
**AGE CLASS**	**Young**	**Adult**	**Young**	**Adult**
**Induction period (min)**	**3.33** / 2.82	**4.11** / 4.51	**4.29** / 4.74	**9.87** / 7.36
**GENDER**	**Female**		**Male**	
**AGE CLASS**	**Young**	**Adult**	**Young**	**Adult**
**Creatinine (μmol/L)**	**97.38** / 98.88	**152.64** / 146.68	**122.29** / 120.30	**165.11** / 167.10
**Total protein (g/L)**	**70.85** / 70.95	**79.20** / 78.70	**79.48** / 79.31	**87n23** / 87.40

Heart rate after 40 minutes and initial rectal temperature were significantly higher in the wild boar captured in corral traps, as well as serum lactate, glucose, potassium, and triglyceride concentrations, whereas HP was significantly higher in wild boar captured in cage traps. Serum AST activity was significantly higher in females than in males. Young wild boar had significantly shorter RP and higher serum ALP activity than adults. IP was significantly longer in the corral-trap captured and adult wild boar than in the cage-trap captured and young ones, respectively. Serum creatinine and total protein concentrations were significantly lower in the female and young wild boar as compared to males and adults (Tables 2, 3 and 4). Higher dosages increased RP and initial respiratory rate, and were negatively correlated with serum enzymatic activity (ALT, ALP, CK, AST, and LDH) and serum lactate, urea and triglycerides concentration. The wild boar with better body condition experienced a shorter IP, and had lower initial respiratory rate, heart rate at 40 minutes, and serum glucose concentration, but showed higher serum CK and LDH activities than the wild boar in lower body condition. Finally, higher environmental temperatures increased IP and initial body temperature, as well as serum sodium, urea, and creatinine concentrations, and decreased serum cholesterol and potassium concentrations (Table 2).

The post-capture activity monitoring of 14 GPS collared wild boar evidenced no mortality for the first 20 days. In fact, no reduction of activity patterns recorded in terms of average speed was observed in the first 10 hours post-capture (262 ± 115 m/h) compared with the same period in the consecutive 3 days (265 ± 144 m/h), according to Mann–Whitney U test (U = 27.5, *p* > 0.05).

## Discussion

### Capture method

Both capture methods, the cage-trap and the corral-trap, were effective and provided good average yield. The higher yield in corral-traps than in cage-traps agrees with previous reports [17]. Nevertheless, the average yield for cage-traps was also higher than one wild boar per capture. There was a high variability among locations, probably due to marked differences in local wild boar abundance, which has been reported to influence capture yield [17].

Mortality fell within the previously reported 1.6%-10.6% range for this species [14,17], and was mostly caused by trauma in corral-traps (4 out of 5 mortality cases recorded). Trauma is a documented cause of mortality when capturing wild boar with corral traps [17,19]. Therefore, cage traps are considered to be safer than corral traps. Hyperthermia is a well-known cause of stress-related mortality in wild ungulates and particularly in wild boar, which are especially prone to hyperthermia when exposed to high environmental temperatures [7,15,46]. Moreover, an increase of activity before immobilization can lead to the production of heat in the muscle and severe elevation of body temperature [47].

Although only operator-activated methods are considered truly selective [48,49], species-specificity for the capture methods used in this study was high (96.3%), likely due to the use of camera traps, which allowed activating the traps once they were regularly visited by wild boar. Blind activation of the traps would produce earlier captures of wild boar, increasing efficiency, but would decrease specificity in turn. Concerning the age-selectivity of the study method, a greater proportion of young wild boar was captured in corral traps, due to the higher rate of capture of family groups (adult female with progeny), as previously reported [11,19].

### Anaesthesia

The anaesthetic protocol used (2.9 mg/kg of TZ and 0.05 mg/kg of M) had a high anaesthetic efficiency (97.6%), higher than the previously reported 55% - 78% range obtained with other anaesthetic protocols used in wild boar [14,15,17,41]. Moreover, it allowed the use of low volumes of drug, which has economic and practical interest as makes the protocol suitable for teleanaesthesia [50].

Anaesthetic induction was quick (4.5 minutes), shorter than the previously reported 5–10 minutes for a combination of 5 mg/kg of TZ and 0.025 mg/kg of M [29] or the 5 minutes reported for a combination of 3.2 mg/kg of TZ and 1.6 mg/kg of X [41], both in feral hogs, but slightly longer than the 3.3 minutes reported in wild boar with a higher dose (used for prolonged surgical procedures) of 5 mg/kg of TZ and 0.1 mg/kg of M [20]. The period of hypnosis allows the safe handling of animals, an appropriate duration of this period is required and varies depending on the specific procedures to perform. The mean HP (61.6 min) was longer than the period of 52 minutes obtained using TZ and X [41], or the 37.6 minutes reported for TZ alone [14]. Anaesthetic recovery is critical in wild boar [13,14,28,51]. For instance, it may be extended by residual activity when using ketamine [13,28] or TZ [14] alone. The addition of M, an α_2_-adrenergic agonist (alternatively X or romifidine may be used) reduced the required TZ dose, providing an anaesthetic RP much shorter (12.8 minutes), than the 43 minutes reported for the aforementioned combination of 3.2 mg/kg of TZ and 1.6 mg/kg of X in feral pigs [41]. In addition, atipamezole (an α_2_-adrenergic antagonist) was effective reversing the anaesthetic effects of M [52]. Reversal of M anaesthesia by atipamezole might uncover residual cyclohexamine effects if the antagonist is administered too early or at tiletamine high dose [53]. However, no such side effects were observed in this study. Further studies are needed to properly assess the efficacy and safety of anaesthetic reversal in anaesthetized wild boar.

The decreasing trends observed in heart rate, respiratory rate, and body temperature have been previously reported in domestic pigs using TZ combined with M [54] or X [54-56]. The oxygen saturation values registered coincide with those previously reported in anaesthetized wild boar, feral pigs, and peccaries and are comparable to the 93.2% SpO_2_ considered indicator of good physiological condition during anaesthesia in these pig species [41,57,58]. Therefore, the anaesthetic protocol used seemed to be efficient and low risk.

Regarding activity patterns, no signs of movement restriction due to anaesthesia were evidenced during post-release monitoring. However, the risk of secondary narcosis in the first hours after handling could not be evaluated in practice due to the low activity of wild boar during daytime [59] and the time rate of fixing positions (1 hour).

### Factors affecting anaesthesia

Anaesthetizing free-ranging wild animals is always a risk, since no preanaesthetic evaluation can be properly performed (even estimating the weight is challenging), and several factors, either external (like trap type, environmental temperature, preanaesthetic stress, dose) or internal (gender, age, body condition) modulate individual response [60]. All these factors were significant in the present study.

According to the best statistical model obtained, the main factor affecting anaesthetic efficacy and stress indicators is trap type. The higher IP, heart rate at 40 minutes, initial rectal temperature, and serum lactate, glucose, potassium, and triglyceride concentrations and the shorter HP shown by the wild boar captured with corral traps indicate that they are more stressful for the wild boar during the preanaesthetic period than cage-traps, decreasing anaesthetic efficiency and animal welfare. That agrees with the higher mortality due to trauma experienced in this type of trap, and was probably related to the physical exercise before capture, since both lactate and potassium increases through anaerobic metabolism due to physical exercise [61,62] and are indicators of capture myopathy [46]. Larger traps have already been reported to cause a higher stress and injuries in captured feral pigs [19]. Preanaesthetic stress is inversely related to the anaesthetic efficacy, requiring higher doses to achieve the same anaesthetic effect and causing dosage inefficacy [17,18,41]. We can not discard that the early darting of animal with blow pipe (HPI) before handling could influence itself animal reaction, but this was probably mediated by its association with the capture system, since it took more time, in average, darting animals in corral traps.

Age-related differences between young (<25 months) and adult (>25 months) wild boar in anaesthetic periods (IP and RP) and physiological variables (serum creatinine and total protein concentrations and ALP activity) are probably related to metabolic differences, since anaesthetic metabolism has been reported to be faster in young animals [63], therefore decreasing IP and RP. Regarding serum biochemistry, adults have higher serum protein concentration [64] and, since serum creatinine is directly related to muscular mass [65], also higher serum creatinine concentration than young animals, as previously reported in other wild ungulate species [66,67]. Higher ALP activities in young animals due to increased bone isoenzyme have been repeatedly reported in wild ungulates [68,69]. The higher serum creatinine and total protein concentrations and AST activity observed in the female wild boar as compared to males suggest a higher stress level in females. AST is a nonspecific but sensitive marker of soft tissue damage [69], whereas creatinine is directly related to muscular mass, and therefore it would be expected to be higher in males, but it may also increase due to renal vasoconstriction induced by catecholamines [65,70]. Higher creatinine levels in females, suggesting a higher adrenergic stress response in this gender, have been reported in other wild ungulate species [71]. Increases in AST and creatinine are related to myopathy and renal vasoconstriction, respectively, which are relevant in the pathogenesis of the four capture myopathy syndromes [46,72]. Nevertheless, and since other stress indicators, like body temperature and other serum enzymatic activities, did not indicate this possible higher stress in females, these results should be considered with caution.

The effects of increasing dosages (longer RP and higher initial respiratory rate, and lower serum enzymatic activity and serum lactate, urea, and triglyceride concentrations) suggest that the wild boar receiving a higher dose experienced less stress, although the longer RP could induce a more intense stress which would be undetected, since monitoring took place during HP. A longer RP with increasing doses has been reported both for wild boar and feral pigs [14,41]. The benefits (lower stress) and risks (longer RP) of higher doses should be counterbalanced for each situation when anaesthetizing wild boar in the field.

The effects of good body condition on anaesthetic variables (shorter IP and lower initial respiratory rate and heart rate at 40 minutes), as well as the lower serum glucose concentration, could be explained by a lower plane of body metabolism in wild boar with a higher percentage of body fat [73], as previously reported in feral pigs [41]. Higher serum CK and LDH activities in the wild boar in good body condition could correspond to their greater body size and amount of tissue releasing these enzymes.

Finally, the effects of high temperatures on both anaesthetic variables (longer IP and higher initial body temperature) and serum biochemistry (higher sodium, urea, and creatinine and lower cholesterol and potassium concentrations) indicate dehydration before anaesthesia, which could lead to heat stroke and to an increased risk of developing capture myopathy [46]. Wild boar are especially prone to hyperthermia when exposed to high environmental temperatures [7], and an increase of activity before immobilization can lead to the production of heat muscle and severe elevation of body temperature. Under these circumstances, immediately cooling the animal with cold water, alcohol or ice packs [47] is paramount to enhance welfare and decrease the probability of adverse anaesthetic consequences, thus, increasing survival rate.

## Conclusion

Both, cage and corral traps are efficient methods to capture wild boar. Cage traps are safer, as demonstrated by mortality rates as well as anaesthetic, physiological, and serum biochemical responses. Nevertheless, mortality fell within the lower range of previously reported data and no additional mortality was registered during post-release monitoring. The anaesthetic combination used (2.9 mg/kg of TZ and 0.05 mg/kg of M) is efficient and safe to immobilize physically captured wild boar, and the addition of an α_2_-adrenergic agonist provides suitable analgesia, muscle relaxation and recovery. Trap type, preanaesthetic stress, anaesthetic dose, gender, age, body condition, and environmental temperature affected anaesthetic efficiency and animal welfare. All these factors must be taken into account when anaesthetizing free-ranging wild boar. In conclusion, this anaesthetic protocol is useful for prolonged handling of wild boar and allows sampling and collecting data for ecological and epidemiological studies.

## Abbreviations

M: Medetomidine; X: Xilacine; TZ: Tiletamine-zolazepam; HPI: Human presence until injection; IP: Induction period; HP: Hypnosis period; RP: Recovery period; ALT: Serum alanine aminotransferase; AP: Alkaline phosphatise; AST: Aspartate aminotransferase; CK: Creatine kinase; LDH: Lactate dehydrogenase; GPS: Global position system; GSM: Global system for mobile communications; GLMz: Generalized linear models; SD: Standard deviation.

## Competing interests

The authors declare that they have no competing interests.

## Authors’ contributions

JAB and JV designed and carried out the study. JV and JRLO provided guidance on anaesthetic and statistical aspects of the study. JAB, BB and JV were involved in field work and data collection. JAB, JV, JRLO, BB and CG assisted in data interpretation and drafting the manuscript. All authors contributed to the critical revision of the manuscript for important intellectual content and have seen and approved the final draft.
